# Measurement of Voice Onset Time in Maxillectomy Patients

**DOI:** 10.1155/2014/925707

**Published:** 2014-01-20

**Authors:** Mariko Hattori, Yuka I. Sumita, Hisashi Taniguchi

**Affiliations:** ^1^Clinics for Oral and Maxillofacial Rehabilitation, University Hospital, Faculty of Dentistry Tokyo Medical and Dental University, Yushima 1-5-45, Bunkyo-ku, Tokyo 113-8549, Japan; ^2^Department of Maxillofacial Prosthetics, Maxillofacial Reconstruction and Function, Division of Maxillofacial/Neck Reconstruction, Graduate School, Tokyo Medical and Dental University, 1-5-45 Yushima, Bunkyo-ku, Tokyo 113-8549, Japan

## Abstract

Objective speech evaluation using acoustic measurement is needed for the proper rehabilitation of maxillectomy patients. For digital evaluation of consonants, measurement of voice onset time is one option. However, voice onset time has not been measured in maxillectomy patients as their consonant sound spectra exhibit unique characteristics that make the measurement of voice onset time challenging. In this study, we established criteria for measuring voice onset time in maxillectomy patients for objective speech evaluation. We examined voice onset time for /ka/ and /ta/ in 13 maxillectomy patients by calculating the number of valid measurements of voice onset time out of three trials for each syllable. Wilcoxon's signed rank test showed that voice onset time measurements were more successful for /ka/ and /ta/ when a prosthesis was used (*Z* = −2.232, *P* = 0.026 and *Z* = −2.401, *P* = 0.016, resp.) than when a prosthesis was not used. These results indicate a prosthesis affected voice onset measurement in these patients. Although more research in this area is needed, measurement of voice onset time has the potential to be used to evaluate consonant production in maxillectomy patients wearing a prosthesis.

## 1. Introduction

Maxillectomy patients often have speech impairments caused by communication between the nasal and oral cavities and a missing palate and teeth, which are important for articulation. Speech rehabilitation includes fitting with a maxillofacial prosthesis and speech evaluation [1, 2]. The intelligibility of these patients is typically evaluated by a listening test [1, 2], but objective evaluation by acoustic analysis is also needed [3, 4]. Acoustic analysis adds important objective and quantitative information to the clinical speech evaluation. Sumita et al. [4] evaluated the speech of maxillectomy patients using vowel formant analysis, while Prunkngarmpun et al. [3] used a nasometer. Chowdhury et al. [5] introduced psychoacoustic evaluation of the syllable /sa/ with these patients. However, it is difficult to conduct detailed assessments of other consonant articulations. An automatic speech test using computerized speech recognition has also been used, although this computer-based evaluation was found to be appropriate only for overall speech assessment in maxillectomy patients, not for detailed consonant assessments [6].

Voice onset time (VOT) is defined as the length of time between the release of a stop and the onset of vocal fold vibration [7]. VOT has been used in the field of phonetics to study language acquisition in healthy individuals [8–11]. Because VOT characterizes consonant articulation, it has also been used in dental studies. Although Ichikawa et al. [12] studied the influence of wearing a palatal plate on VOT in four normal subjects and Akpinar et al. [13] studied changes in VOT before and after soft palate implant surgery, VOTs in maxillectomy patients have yet to be determined. One reason for this is that, due to air leakage through the nose, maxillectomy speech is characterized by formant impairment [4] and low energy consonant production [5]. Formant impairment makes it difficult to detect onset of vocal vibration, and low energy consonant production makes it difficult to detect the burst point. As a result, VOT measurement is considered challenging in these patients. This study aimed to establish VOT measurement criteria for maxillectomy patients to evaluate their speech. First, we established criteria for the VOT evaluation of these patients with and without a prosthesis. The null hypothesis of this study is that VOTs for /ka/ and /ta/ are equally measurable in maxillectomy patients with and without wearing a prosthesis.

## 2. Materials and Methods

### 2.1. Subjects

Participants were 13 patients who visited the Clinics for Oral and Maxillofacial Rehabilitation, University Hospital, Faculty of Dentistry Tokyo Medical and Dental University, in 2006. Inclusion criteria were having a maxillary defect due to maxillary tumor resection and wearing an obturator prosthesis fabricated at the clinic. Exclusion criteria were having oral pain or other problems with the prosthesis within 1 month of placement and having auditory abnormalities. Patients (4 men, 9 women; mean age and range, 59.8 and 52–76) provided informed consent to participate in speech testing with and without their prosthesis. Patients' gender, age, and defect classification according to Aramany's classification [14] are in Table 1. This study was approved by the Ethics Committee of the Faculty of Dentistry Tokyo Medical and Dental University (Approval no. 215).

### 2.2. Method

In a soundproof room, a microphone (F-VX400; Sony Co., Tokyo, Japan) was placed 15 cm from the patient's mouth. The patient then read sentences from the fairy tale *Jack and the Beanstalk* in which /ka/ and /ta/ occurred three times. We saved these sentences onto a computer hard drive using the sound interface Sound Blaster Extigy (Creative Technology Ltd., Creative Resource, Singapore) and conducted a spectral analysis of those consonants using Sugi Speech Analyzer software (Animo Co., Yokohama, Japan).

To measure VOT, we analyzed voice samples using Fast Fourier Transform (512 points, 16 bits) with a 16000 Hz sample rate to generate narrowband (with a filter bandwidth of 45 Hz) and broadband (300 Hz) spectrograms. The Hamming window was used as the window function.

### 2.3. Measurement Criteria for the Voice Onset Measurement in Patients

**Figure 1:**
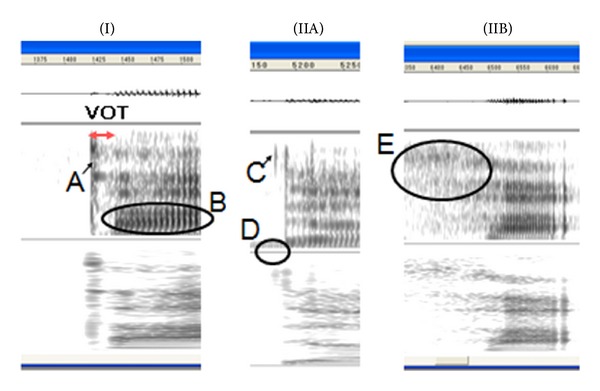
Spectrograms of normal and atypical production of stop consonants. (I) Spectrogram of a normally produced stop voiceless consonant, where A is the consonant burst and B is vocal fold vibration. (IIA) Spectrogram of a consonant showing a negative VOT, where C is a consonant burst and D is vocal fold vibration that started earlier than the consonant onset. (IIB) Spectrogram with an unclear consonant onset (E).

The onset of a consonant was defined as the VOT start point, and the appearance of clear pitch was defined as the VOT endpoint. When the appearance of pitch was not obvious, the appearance of the first and second formants implied the initiation of a vowel. In Figure 1, the left spectrogram shows a typical sound spectrum of a stop consonant in normal subjects.

To conduct VOT measurement in maxillectomy patients, we established measurement criteria for each consonant to allow for the possibility of observing spectra not typically seen in normal individuals.

Because /ka/ and /ta/ start with voiceless plosives, VOT measurement was considered invalid due to mispronunciation when characteristics of other consonants (e.g., voiced consonants or fricatives) were apparent. For example, when the VOT value was 0 or negative, we considered the measurement invalid. In Figure 1, the middle spectrogram shows a negative VOT due to the clear presence of a voiced sound before the onset of the consonant. Also, a measurement was judged invalid when a voiceless part was unclear prior to the onset of a consonant or when the starting boundary of noise components was not clear. In Figure 1, the spectrogram on the right side shows unclear onset of a consonant.

### 2.4. Statistics

For each syllable, we calculated the number of valid VOT measurements out of three trials. Wilcoxon's signed rank test was used to analyze significant differences in the number of valid VOT measurements out of three trials (dependent variable) under two different conditions (with and without a prosthesis). The analysis was performed using SPSS 13.0J for Windows (SPSS Inc., Chicago, IL). Significance was set at *P* < 0.05.

## 3. Results and Discussion

Table 2 shows the number of successful VOT measurements out of three trials. When a prosthesis was not worn during testing, the median and range were 2 (0–3) for /ka/ and 2 (0–3) for /ta/, respectively. When a prosthesis was worn, the corresponding values were 3 (3-3) for /ka/ and 3 (1–3) for /ta/. Wilcoxon's signed rank test showed significant differences in the number of successful VOT measurements for both /ka/ (*Z* = −2.232, *P* = 0.026) and /ta/ (*Z* = −2.401, *P* = 0.016). For both syllables, the number of successful VOT measurements was larger with a prosthesis.

VOT measurements were invalid in many of the maxillectomy patients when not wearing a maxillofacial prosthesis, indicating that their production of plosives such as /ka/ and /ta/ was impaired by maxillary deficiency. On the other hand, VOT measurements were highly successful in the presence of a prosthesis. Thus, we reject the null hypothesis of this study. We believe VOT measurements were more successful in patients with a prosthesis because the obturator blocking the passage between the oral and nasal cavities allowed the vocal tract to close, and the artificial teeth and palate in the denture base facilitated pronunciation. Thus, the prosthesis allowed the patients to correctly produce consonants. Our results demonstrate that VOT measurement can be used to evaluate consonant production in maxillectomy patients wearing a prosthesis.

Nasalance and formant measurements to evaluate nasal outflow and vowel distortion, respectively, have also been used to objectively assess speech intelligibility in maxillectomy patients [3, 4]. These measurements have often been performed in patients without a prosthesis. Past research indicates that computer-based speech testing is appropriate for patients exhibiting a severe speech disorder (e.g., patients without a prosthesis) [6]. However, the present study demonstrated the usefulness of VOT measurement for detailed evaluation of consonant articulation, particularly in patients with a prosthesis. In fact, VOT measurements can reveal the mechanism of consonant unnaturalness in maxillectomy patients. With further research, changes in VOT might explain the difference in psychoacoustic features between maxillectomy speech and normal speech [5].

We plan to perform VOT measurements of other consonants on a large number of patients to further define VOT measurement criteria, use the criteria to investigate the effect of prosthesis design on speech, and evaluate consonant articulation before and after prosthesis adjustment. In this study, we successfully established and applied VOT measurement criteria to evaluate VOT in the speech of maxillectomy patients. The results suggest that VOT measurements in these patients are more successful when a prosthesis is used. This finding is important clinically because it implies the future potential of VOT measurement for investigating the influence of prosthesis design and fit on consonant articulation.

## 4. Conclusion

VOT measurements in 13 maxillectomy patients were more successful for /ka/ and /ta/ when a prosthesis was worn. The results indicate that wearing a prosthesis affects voice onset measurements in maxillectomy patients. Thus, VOT measurement has the potential to be used to evaluate consonant production in maxillectomy patients with a prosthesis.

## Figures and Tables

**Table 1:** Patient characteristics.

Patient number	Sex	Age	Aramany's classification
1	F	62	I
2	F	60	II
3	F	75	II
4	M	54	II
5	M	52	II
6	F	71	II
7	F	60	II
8	F	70	I
9	M	61	II
10	M	59	IV
11	F	76	II
12	F	65	IV
13	F	65	II

**Table 2:** Number of successful VOT measurements of /ka/ and /ta/ with and without a prosthesis in 13 maxillectomy patients.

Patient number	Number of successful measurements			
	Without prosthesis		With prosthesis	
	/ka/	/ta/	/ka/	/ta/
1	3	0	3	3
2	3	2	3	3
3	3	3	3	3
4	1	2	3	3
5	2	0	3	3
6	3	1	3	1
7	0	3	3	3
8	3	3	3	3
9	0	1	2	1
10	3	2	3	3
11	2	3	3	3
12	1	0	3	3
13	3	1	3	3
